# Bone Mineral Density, Serum Calcium, and Vitamin D Levels in Adult Thalassemia Major Patients: Experience From a Single Center in Eastern India

**DOI:** 10.7759/cureus.26688

**Published:** 2022-07-09

**Authors:** Soumya Santra, Kunal Sharma, Ipsita Dash, Shaikat Mondal, Himel Mondal

**Affiliations:** 1 Pharmacology, College of Medicine and JNM Hospital, Kalyani, IND; 2 Pharmacology, Government Medical College, Haldwani, IND; 3 Biochemistry, Saheed Laxman Nayak Medical College and Hospital, Koraput, IND; 4 Physiology, Raiganj Government Medical College And Hospital, Raiganj, IND; 5 Physiology, Saheed Laxman Nayak Medical College and Hospital, Koraput, IND

**Keywords:** dexa scan, hematology disorders, eastern india, serum calcium, osteopenia, vitamin d, thalassemia, osteoporosis, cholecalciferol, bone mineral density

## Abstract

Background and objective

Patients suffering from thalassemia major are at higher risk of osteoporosis. Due to their decreased life expectancy, the number of adult patients is low. However, their bone health is rarely checked in developing countries like India. There is no data available in the literature on the bone mineral density (BMD) of adult (aged ≥18 years) thalassemia major patients in eastern India. In this study, we aimed to measure the BMD and serum calcium and vitamin D levels in adult thalassemia major patients and to compare them with healthy controls.

Materials and methods

We conducted this cross-sectional observational study at a tertiary care hospital in eastern India. We recruited adult thalassemia major patients who were not on calcium or vitamin D supplements. Their BMD was measured by dual-energy X-ray absorptiometry (DXA) on the lumbar spine (L1-L4). Venous blood was tested for serum calcium and vitamin D levels. We compared the parameters between the cases and controls by using the Mann-Whitney U test.

Results

A total of 31 (male = 19, female = 12) patients with a median age of 28 years comprised the case group. Age- and sex-matched controls showed similar height but higher weight and BMI. The serum calcium level was similar (p = 0.43) in the case and control groups but T-score (p = 0.0003) and vitamin D levels (p: <0.0001) were significantly lower in thalassemia major patients.

Conclusion

Based on our findings, adult thalassemia major patients have lower BMD and vitamin D levels. Although the serum calcium may be normal in these patients, they should still be screened both for BMD and vitamin D for prompt and early detection of risks and complications so that a proper management strategy can be implemented.

## Introduction

The first case of thalassemia in India was reported in 1938. Since then, knowledge about its pathophysiology and treatment modalities has evolved. The availability of blood transfusion, chelation therapy, and other supplementary treatment facilities have steadily improved the quality of life for thalassemia patients [1]. South Asia accounts for approximately 23% of global hemoglobin disorders with an estimated 17 million β-thalassemia patients in India alone [2].

The life expectancy among β-thalassemia patients has increased significantly in recent years [3]. Consequently, the incidence of treatment-related disorders, especially osteoporosis, has also increased. Several underlying mechanisms can lead to a misbalance between the activity of osteoblasts and osteoclasts, which leads to decreased bone mineral density (BMD) [4,5].

Meena et al. measured the BMD of Indian patients (aged between 2-18 years) with thalassemia major and reported reduced BMD in this group of patients [6]. Similarly, Singh et al.'s study has revealed that about 80% of North Indian adolescent and young adult thalassemia major patients suffer from vitamin D insufficiency while 42% have osteoporosis [7]. Merchant et al. conducted a study in Mumbai involving patients aged 10-25 years and found that 80% of thalassemia major patients have osteoporosis [8]. These studies either had adolescents or young adults as the sample. To the best of our knowledge, there is no study in the literature regarding the BMD of adult (aged ≥18 years) thalassemia major patients from eastern India.

In light of this, we aimed to conduct this study to assess the risk of osteoporosis among adult thalassemia major patients in eastern India. In order to quantify and analyze this risk, we aimed to measure the serum calcium and vitamin D levels and BMD T-scores in these patients.

## Materials and methods

Ethical approval

This study involved adult (aged ≥18 years) research participants who were capable of providing written consent for their participation. We conducted the study by following the guidelines laid down by the World Medical Association Declaration of Helsinki, updated in 2013. The local institutional ethics committee approved the study (Number: REF-10/IEC, dated 14/08/2019).

Study design and setting

We conducted this cross-sectional observational study at a tertiary care hospital in eastern India from August 2019 to January 2020. We compared the measured parameters between the cases and their age and sex-matched healthy controls. The research participants were recruited from the outpatient department of Medicine (Thalassemia Clinic) at the tertiary care center.

Sample size

No similar study involving adult thalassemia patients has been conducted in this geographical region. Hence, after reviewing relevant literature on other age groups, as well as regional and international data, we calculated the minimum sample size with α = 0.05, β = 0.2 (i.e., power of the study = 80%), expected BMD in cases as 0.48 and that in controls as 0.57, with an expected standard deviation of 0.11 [6]. The calculated sample size was 24 each in both the case and control groups. However, for a scalable sample size for the t-test, we aimed to recruit 30 research participants to be included in each group.

Sampling method

**Figure 1:**
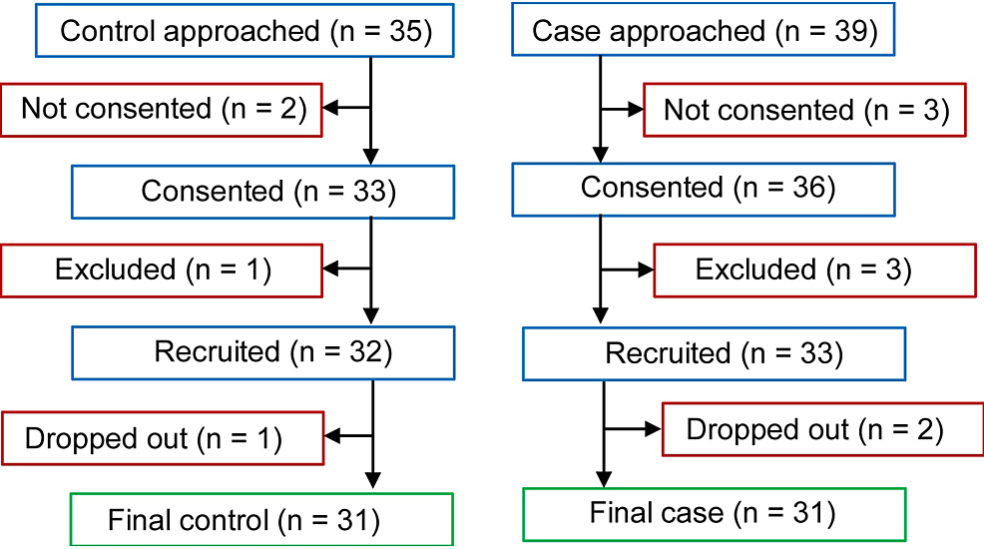
Flow chart detailing the recruitment of controls and cases for the study

We used a convenience sampling method for this study. Consecutive patients attending the Thalassemia clinic who met the inclusion criteria and were not excluded based on pre-fixed criteria were recruited for the study. Adult (aged ≥18 years) thalassemia major patients (diagnosed based on high-performance liquid chromatography) who provided written consent for participation were included. Patients taking oral calcium or vitamin D, any antiepileptic drugs, or proton pump inhibitors were not recruited. Also, patients with acute illness, any mental illness, or any major physical deformity that rendered height measurement not possible were excluded from the study. After the recruitment of an adequate number of cases, we started the recruitment of age- and sex-matched otherwise healthy controls from patients’ relatives and attendants. A detailed history was taken to rule out any pre-existing disease among controls. However, we did not conduct any laboratory tests to confirm if the individual was healthy from a laboratory-parameter viewpoint. Figure 1 depicts the flow chart that summarizes the recruitment process.

Measurements

The height was measured by a portable stadiometer (PRESTIGE - SM, Hardik Meditech, Sonipat, India) to the nearest 1 mm while the research participants stood straight in the Frankfurt position without shoes or socks and with light clothing. The measurement was taken after a normal exhalation. All the measurements were carried out by a single expert clinician with previous experience in anthropometry. The measurements were conducted between 10 am and 12 pm. The weight was measured using a digital weighing scale (Omron HBF 701 Karada Scan, Krell Precision, Yangzhou, China) having a precision of 100 gm. BMI was calculated using Quetelet's index (weight in kg/height in m^2^). After taking aseptic precautions, blood was collected by venipuncture in a commercial vacutainer and immediately sent to the laboratory for estimation of vitamin D and calcium levels. In the laboratory, vitamin D was measured by electrochemiluminescence immunoassay (ECLIA) (Cobas 6000, Roche, Basel, Switzerland) and serum calcium was measured by the o-cresolphthalein complexone (o-CPC) method. The BMD was measured by dual-energy X-ray absorptiometry (DXA) from the lumbar spine (L1-L4) by the Lunar Prodigy Advance DXA system (GE Healthcare, Chicago, IL). The measurement was done by using a single machine for all the patients and controls to reduce any bias.

Reference ranges of BMD, vitamin D, and serum calcium

The risk of osteopenia and osteoporosis was categorized as per T-scores obtained by BMD according to the reference values as follows: normal: ≥ -1, osteopenia: -1.01 to -2.49, and osteoporosis: ≤ -2.5 [9]. The level of vitamin D was categorized according to the reference values as follows (as per the test method): optimal: 25 - 80 ng/mL, mild-moderate deficiency: 15 - 24.99 ng/mL, and severe deficiency: <15 ng/mL. Serum calcium reference range was estimated according to the test method as follows: 8.8 - 10.6 mg/100 ml.

Statistical analysis

Data are presented in mean, standard deviation, and ranges. Variables between the groups were compared using the Mann-Whitney U test as the data were not normally distributed. Categorical data were tested by the Chi-square test and McNemar’s test [10]. Spearman correlation coefficient and multiple linear regression analysis were applied to find the correlation and predict the capability of variables to estimate the outcome variable. A p-value <0.05 was considered statistically significant. All the statistical tests were carried out in GraphPad Prism 6.01 (GraphPad Software, San Diego, CA) and SPSS Statistics for Windows, Version 20.0 (IBM Corp., Armonk, NY).

## Results

A total of 31 (male = 19, female = 12) adult (median age: 28 years) patients suffering from thalassemia were included in the analysis. All the variables were compared with an age- and sex-matched otherwise healthy control group with 19 males and 12 females with a median age of 28 years. The age of the subjects was similar in both groups as we intentionally recruited controls with similar ages (p: >0.99). The height was also similar in the case and control groups. However, weight and BMI were lower among thalassemia major patients (Table 1).

**Table 1:** Age, anthropometric parameters, vitamin D and serum calcium levels, and T-scores among controls and cases *Statistically significant p-value based on the Mann-Whitney U test

Parameters	Controls (n = 31)		Cases (n = 31)		P-value
	Median (Q1 – Q3)	Range (min – max)	Median (Q1 – Q3)	Range (min – max)	
Age (years)	28 (24 – 30)	18 – 35	28 (24 – 30)	18 – 35	>0.99
Height (cm)	165 (159.5 – 174.5)	153 – 183.5	160 (155 – 166.5)	152 – 174.5	0.09
Weight (Kg)	67.6 (61.7 – 77.9)	50.2 – 109.2	54.6 (45.5 – 63)	29.2 – 79	<0.0001
BMI (kg/m^2^)	24.83 (22.41 – 27.42)	19.26 – 36.8	20.3 (17.71 – 24.12)	10.53 – 30.29	0.0002*
Vitamin D (ng/mL)	37.56 (27.5 – 60.7)	17.5 – 78.7	23.58 (14.1 – 28.3)	9.38 – 56.3	<0.0001
Calcium (mg/dL)	9.7 (9.2 – 10.1)	8.8 – 10.8	9.6 (9.1 – 10.1)	8.4 – 10.8	0.43
T-score	-1 (-1.1 to -0.9)	-2.4 to -0.8	-1.5 (-2.1 to -1)	-4.8 to -0.9	0.0003*

Although the serum calcium levels were similar between the groups (p = 0.43), the vitamin D levels (p: <0.0001) and T-scores (p = 0.0003) were significantly lower in patients suffering from thalassemia major.

The correlation of the T-score versus other variables is shown in Table 2. When we conducted a correlation analysis between the vitamin D levels and T-scores (with negative sign), we found a significant positive correlation in controls. However, the correlation in thalassemia patients was not significant. A similar result was found for the correlation between blood calcium levels and T-scores.

**Table 2:** Correlation coefficient between T-scores versus other variables in controls and cases *Statistically significant p-values rs: Spearman's correlation coefficient; CI: confidence interval

Variables	Control (n = 31)			Cases (n = 31)		
	*r*_s_	95% CI	P-value	*r*_s_	95% CI	P-value
Age (years)	-0.5612	-0.7682 to -0.2478	0.0005*	0.2437	-0.1319 to 0.5582	0.0932
Weight (kg)	-0.1191	-0.4630 to 0.2560	0.2617	0.1006	-0.2733 to 0.4482	0.2951
Height (cm)	0.0155	-0.3504 to 0.3773	0.4670	-0.1191	-0.4630 to 0.2560	0.2618
BMI (kg/m^2^)	-0.1827	-0.5126 to 0.1942	0.1627	0.1392	-0.2367 to 0.4789	0.2276
Vitamin D (ng/mL)	0.4272	0.07482 to 0.6847	0.0083*	-0.09829	-0.4463 to 0.2755	0.2994
Calcium (mg/dL)	0.4769	0.1367 to 0.7165	0.0033*	0.05906	-0.3116 to 0.4141	0.3762

In multiple liner regression analysis among controls, the overall predictor variables including age (years) (p = 0.04), weight (kg) (p = 0.67), height (cm) (p = 0.68), BMI (kg/m^2^) (p = 0.64), vitamin D (ng/mL) (p = 0.78), and calcium (mg/100 mL) (p = 0.03) statistically significantly predicted T-score, F (6, 24) = 3.5, p = 0.01. However, individually, only age and calcium levels showed significant differences.

In multiple liner regression analysis among cases, the predictor variables such as age (years) (p = 0.14), weight (kg) (p = 0.97), height (cm) (p = 0.95), BMI (kg/m^2^) (p = 0.88), vitamin D (ng/mL) (p = 0.78), and calcium levels (mg/100 mL) (p = 0.82) did not predict T-scores in a statistically significant manner, F (6, 24) = 0.55, p = 0.77.

When we categorized the cases and controls according to risk by T-scores, there was a higher risk of osteopenia and osteoporosis among adult thalassemia patients (χ^2^ = 13.97, p = 0.0009). When we categorized the cases and controls according to vitamin D levels, the optimal level of vitamin D in the cases was lower and deficiency was higher (χ^2^ = 14.56, p = 0.0007). When we performed the McNemar test, we found a non-significant association (p = 0.27). However, there may be a risk (OR: 2.25) of osteopenia and osteoporosis when vitamin D level is lower. These auxiliary analyses are available in a supplementary file at https://doi.org/10.6084/m9.figshare.20000726.v1.

## Discussion

In our study to analyze the risk of osteoporosis in adult thalassemia major patients in eastern India, we found that this patient group has higher rates of osteopenia and osteoporosis in comparison to normal healthy controls. Although serum calcium was not deficient in thalassemia patients, their vitamin D levels were lower compared to their healthy counterparts.

For this study, we enrolled a sample aged ≥18 years. However, the findings of this study are corroborative of other Indian studies where either adolescents or a combination of adolescents and adult patients were included as the sample [6-8]. Our results support the findings on BMD in thalassemia patients by Aslan et al. from Turkey [11]; Abbasi et al., Bekheirnia et al., and Shamshirsaz et al. from Iran [12-14]; Domrongkitchaiporn et al. from Thailand [15]; Soliman et al. from Egypt [16]; Vogiatzi et al. from the US [17]; and Tharwat et al. from Saudi Arabia [18].

Blood transfusion and iron chelation are the available principal treatments for thalassemia major [19]. However, it has been found that even with adequate blood transfusion and chelation therapy, the bone mass is lower in thalassemia major patients [17]. Other studies have reported that along with low BMD [13], the blood levels of calcium are also low in thalassemia patients [20]. Hence, early detection and timely therapy with calcium and vitamin D may reduce the chances of these patients developing osteoporosis [21].

It is already known that many factors like age, genetics, sexual maturation, physical activity, lifestyle, hormonal status, and daily calcium intake influence BMD in most normal individuals. It is also estimated that 90% of peak bone mass is reached by the age of 18 years [22,23]. An interesting finding in our study is that the vitamin D level exhibits a positive correlation with T-scores in the control group but not in the case group. Based on our study, we can suggest that the T-score is affected differently in thalassemia cases. We may propose that this may be due to the long-term use of iron chelators, the iron-overload status of various vital organs including the parathyroid gland, ineffective erythropoiesis leading to bone expansion, additives, and anticoagulants used in blood bags [24]. All these factors may lead to altered rates of bone turnover producing changes in the organic and mineral matrix. Thus, we may conclude that age and calcium levels, which are predictors of the T-score in normal individuals, are not to be solely considered predictors of the T-score among thalassemia major cases.

A study by Valderrábano et al. found that the anemia resulting from the depletion of normal hemoglobin levels is not the cause of low BMD [25]. However, a higher rate of erythropoiesis may be responsible for low BMD [26]. Further research has shown that thalassemia patients have high parathyroid hormone levels with a lower level of Insulin-like growth factor (IGF) [16,27]. Hence, hormone replacement therapy with IGF may be beneficial in treating thalassemia major patients who have osteopenia or risk of osteoporosis [16]. However, stakeholders should stress premarital screening for thalassemia as only antenatal screening can prevent the disease, and knowledge of common hemoglobin patterns in a region would help in the detection of thalassemia.

There are several limitations to this study. Although we recruited an adequate number of patients based on our sample size calculation, a higher number of subjects would increase the power of the study and result in more acceptable generalizability. Furthermore, we could not carry out additional tests like complete blood count, reticulocyte count, soluble transferrin receptor, endocrine functions, iron status, level of phosphorous, and parathyroid hormone due to funding limitations. These data could provide us with further insights into the topic. In addition, the BMD test in thalassemia patients poses certain challenges. Pellegrino et al. have mentioned that there are limitations in assessing BMD with DXA scan, especially in the pediatric group of thalassemia major population owing to their disease-related pre-existing characteristic bone morphology and deformities [28]. However, we have concentrated our study mostly on well-managed thalassemia patients who had no orthopedic deformities or complaints. It has also been concluded from the previously published literature that Z-score ≥2.0 does not however rule out chances of pathological fractures and skeletal fragility [29]. Trabecular bone score (TBS) could evolve as a useful tool to subcategorize patients with low BMD [30]. The N-telopeptides and C-telopeptides are used for measuring the bone turnover marker. However, this facility was not available in our settings. For future studies on a similar topic, these should be considered as well.

## Conclusions

The BMD of adult (aged >18 years) thalassemia major patients showed a higher risk of osteopenia and osteoporosis when compared to healthy controls. Although there was no difference in serum calcium levels between the groups, thalassemia patients had lower vitamin D levels. Hence, adult thalassemia major patients should be screened both for BMD and vitamin D for the prompt and early detection of the risk of bone disease and to start appropriate treatment to limit the progress towards osteoporosis. As this was a single-center study with a limited number of patients, further large-scale studies are required to provide more generalizable results.
